# Serious Hazards of Transfusion (SHOT) haemovigilance and progress is improving transfusion safety

**DOI:** 10.1111/bjh.12547

**Published:** 2013-09-14

**Authors:** Paula H B Bolton-Maggs, Hannah Cohen

**Affiliations:** 1 SHOT Office, Manchester Blood Centre and University of ManchesterManchester, UK; 2 Department of Haematology, University College London Hospitals NHS Foundation Trust and University College LondonLondon, UK

**Keywords:** haemovigilance, transfusion safety, Serious Hazards of Transfusion, transfusion errors, transfusion reactions

## Abstract

The Serious Hazards of Transfusion (SHOT) UK confidential haemovigilance reporting scheme began in 1996. Over the 16 years of reporting, the evidence gathered has prompted changes in transfusion practice from the selection and management of donors to changes in hospital practice, particularly better education and training. However, half or more reports relate to errors in the transfusion process despite the introduction of several measures to improve practice. Transfusion in the UK is very safe: 2·9 million components were issued in 2012, and very few deaths are related to transfusion. The risk of death from transfusion as estimated from SHOT data in 2012 is 1 in 322 580 components issued and for major morbidity, 1 in 21 413 components issued; the risk of transfusion-transmitted infection is much lower. Acute transfusion reactions and transfusion-associated circulatory overload carry the highest risk for morbidity and death. The high rate of participation in SHOT by National Health Service organizations, 99·5%, is encouraging. Despite the very useful information gained about transfusion reactions, the main risks remain human factors. The recommendations on reduction of errors through a ‘back to basics’ approach from the first annual SHOT report remain absolutely relevant today.

Blood transfusion is very safe and effective when used appropriately. Questions about the safety of blood transfusion arose partly as a result of the observation of transfusion-transmitted infections in heavily transfused haemophilia patients. The early ‘haemovigilance’ schemes for patients registered in haemophilia centres across the UK in the late 1970s provided valuable information about the benefits and complications of treatment, particularly viral infections. At that time there was no systematic surveillance in the UK for complications of blood component transfusion.

Concerns about wrong transfusions due to error were confirmed by a survey of 400 haematology departments in 1991. The 245 respondents identified 111 wrong blood incidents, with six deaths and 12 instances of major morbidity due to ABO incompatibility (McClelland, 1998). Some also reported ‘near miss’ events that had not been part of the questionnaire. McClelland (1998) recommended that there be a national reporting scheme for critical transfusion incidents and near misses. ‘Near miss’ events are defined as any error, which if undetected, could result in the determination of a wrong blood group or transfusion of an incorrect component, but was recognized before the transfusion took place. For example, one event reported to SHOT in 2011 (wrong labelling of two patients' samples taken at the same time, labelled away from the patients), resulted in one ABO-incompatible transfusion with haemolysis, and one ‘near miss’ because the second patient had a historical blood group so the error for that patient was detected and wrong transfusion avoided (Bolton-Maggs & Cohen, 2012, page 27). The need for formal surveillance of blood transfusion for both errors and pathological incidents was clear.

There were several contributory factors leading to the development of a national surveillance scheme for transfusion. The Public Health Laboratory Service, working together with haematologists and transfusion specialists, was developing surveillance for transfusion-transmitted infection. The Medical Directors of the National Blood Services in England and Scotland met to discuss the implications of the European parliament resolutions on blood safety, which would set standards for transfusion supplies (European Commission, 2005). A working party was set up in 1994 to produce proposals for a UK-wide surveillance scheme for reporting transfusion complications. The Serious Hazards of Transfusion (SHOT) voluntary reporting scheme was launched in 1996 (Williamson *et al*, 1996) with the support of 8 Royal Colleges and 6 other professional bodies and is funded by the four national Blood Services. SHOT became affiliated to the Royal College of Pathologists in 1997 and its activities are overseen by a steering group whose membership includes representatives from the Royal Colleges (medical and nursing) and other specialist societies.

Transfusion is a complex multistep process involving members of several different professional groups, nurses, doctors, laboratory scientists as well as the donors and recipients. The many steps result in several risk points (Table 1). At each of these steps mistakes may be made that put patients' lives at risk. These result from omission of essential checks (short cuts) and perhaps an assumption that someone else is responsible for safety.

**Table 1:** Hotspots for errors in the transfusion process: multiple steps and many different professional groups

Location	Critical point	Health care professional
Blood donor centre or session	Identification of donor. Assessment of donor for safety of donation Identification of donation	Donation session staff
Blood centre	Processing and issue	Blood centre laboratory staff
Ward or outpatient clinic	Assessment of recipient and decision to transfuse	Medical and nursing staff
Ward	Prescription Request form	Medical staff Medical and nursing staff
Ward or phlebotomy clinic	Sampling for pretransfusion testing Transfer of sample to laboratory	Doctors, midwives, nurses, phlebotomists Porters
Laboratory	Reception, testing, allocation of component, labelling and issue	Medical laboratory assistants, Biomedical scientists
Blood transfusion laboratory or remote blood refrigerator	Collection from storage site	Porters, nursing staff
Ward, operating theatre, emergency department	Bedside administration checks Monitoring or adverse incidents	Nurses, midwives, doctors, operating department practitioners

By 1996 the risk of infection from transfusion was already extremely low, 1 in 500 000 for human immunodeficiency virus (HIV), 1 in 60 000 for hepatitis B virus (HBV) and 1 in 100 000 for hepatitis C virus (HCV) (Williamson *et al*, 1996). These low rates resulted from a combination of donor exclusion and screening of donations for these particular viruses. However, the risk of receiving the wrong blood was considerably higher, estimated to be 1 in 30 000 (Love & Lowe, 1998). These risks remain very small, but real (Table 2) (Public Health England, 2013), and the greatest risk to patients remains that an error will be made in the process, most often at the point of blood sampling, in the laboratory, or at bedside administration. This may result in transfusion of a wrong component, at worst an ABO incompatible red cell transfusion leading to major morbidity or death. The objectives for SHOT are for the findings to be used to improve standards of hospital transfusion practice, educate users on transfusion hazards and their prevention, aid production of clinical guidelines in blood transfusion and inform national policy on transfusion safety. In 2013, with publication of the 16th Annual SHOT Report (Bolton-Maggs *et al*, 2013a), we can ask whether these objectives have been achieved and whether there is evidence that transfusion is safer. An additional question is whether it is realistic to expect blood transfusion ever to become completely safe. It is sobering to read the summary of recommendations from the 1996/7 report because they are not different from those of 2012, focusing on correct identification of the patient at the time of sampling and at the time of transfusion, and noting that the bedside check is ‘vital in preventing transfusion error’ (Love & Lowe, 1998).

**Table 2:** Estimated risk of infection from transfusion in the UK (Public Health England, 2013) and risk of major morbidity or death (all causes) from transfusion based on SHOT data for 2012 (Bolton-Maggs *et al*, 2013a)

Category	Risk per million donations [95% confidence interval] for viral infections, and per million components issued for SHOT data	Reciprocal expression of same risks, 1 per number of components issued
Major morbidity	46·7 (all causes)	1 in 21 413
Death	3·1 (all causes)	1 in 322 580
Hepatitis B	0·76 [0·22–1·61]	1 in 1·3 million
Hepatitis C	0·036 [0·015–0·07]	1 in 28 million
HIV	0·15 [0·09–0·32]	1 in 6·7 million

## The development of haemovigilance

Haemovigilance is a relatively recent development in transfusion safety. It is defined as surveillance procedures covering the whole transfusion chain, from collection of blood and its components to follow-up of recipients, intended to collect and assess information on unexpected or undesirable effects resulting from the therapeutic use of labile blood products and to prevent their occurrence or recurrence (International Haemovigliance Network [IHN], 2012). The aims are to identify trends in adverse reactions and events, thereby to inform transfusion policy, to target areas for improvement in practice, to stimulate research, to raise awareness of transfusion hazards, to be an early warning of new complications and to improve safety of transfusion for patients.

## Haemovigilance in other countries

### Europe

Haemovigilance systems were also developed in other countries, predominantly in Europe, as a result of the impact of HIV and hepatitis transmissions. The term ‘haemovigilance’ was probably first used in France in 1991, and set into law by 1993 mandating the reporting of all adverse events in recipients. This legal framework included a requirement to trace each unit from donor to recipient or disposal. Subsequent European Union (EU) legislation, the EU Blood Safety Directives (European Commission, 2003, 2005) resulted in the development of haemovigilance in many countries, often based on the SHOT scheme. As a result of EU legislation, all member states are required to report serious adverse reactions and events annually via their ‘competent authority’, which for the UK is the Medicines and Healthcare products Regulatory Authority (MHRA). The EU legislation was transcribed into UK law [the UK Blood Safety and Quality Regulations (BSQR, 2005)] and requires reporting of all serious adverse reactions (such as haemolysis or infection) but also serious adverse events caused by a process failure in the quality management system within the Blood Establishment or the hospital transfusion laboratory, regardless of whether the component was transfused (SHOT-reportable as incidents if transfused and generally ‘near miss’ events if not). Adverse events occurring in the clinical area without laboratory quality issues are not EU reportable but are reportable to SHOT and constitute the largest sub-groups of SHOT reports (including episodes of avoidable, delayed or undertransfusion, ‘near miss’ events, incidents where the patient received a correct transfusion despite errors — ‘right blood right patient’, and mistakes with anti-D immunoglobulin administration to RhD negative women during and after pregnancy).

The system in the Netherlands, ‘Transfusion Reactions in Patients' (TRIP), has reported annually since 2002 with 96% of hospitals participating in 2011 (2601 reports; TRIP Annual Report, 2011) and an overall reporting rate of 3·9 reports per 1000 components issued. Variable reporting rates reflect both the ability to comply (staff, education) and also the extent to which reactions or clinical events are recognized as being related to a transfusion.

### North America

In the USA, the Food and Drug Administration (FDA) requires reporting of serious adverse reactions and deaths. From 2008 to 2012 198 deaths were reported (US Food and Drug Administration, 2013) (Table 3). The most common cause of transfusion-related death was transfusion-related acute lung injury (TRALI) in 74 recipients. Reduction in the annual rate of TRALI is attributed to changes in transfusion practice (as in the UK) including reduction in plasma components from female donors. Thirteen of 21 deaths from bacterial infection were caused by platelets. The estimated components transfused per annum are 24 million (compared with about 3 million in the UK) and the FDA indicates that these numbers are unlikely to reflect complete participation.

**Table 3:** Transfusion-related deaths reported to the FDA 2008–2012 (US Food and Drug Administration, 2013)

Complication	Total (*n*)	%
Transfusion-related acute lung injury	74	37
Haemolytic transfusion reactions (non-ABO)	31	16
Haemolytic transfusion reactions (ABO)	22	11
Microbial infection	21	11
Transfusion-associated circulatory overload	35	18
Anaphylaxis	12	6
Other	3	1
	198	100

The United States Center for Disease Control has developed a voluntary and anonymous national reporting scheme with internet-based entry (a Hemovigilance Module in the National Healthcare Safety Network). By 2012 more than 140 institutions had enrolled, estimated to reflect surveillance of <5% of transfusions. Baseline data collected by the National Blood Collection and Utilization Survey (NBCUS) for 2008 suggested that the reported adverse reaction rate was 2·6 events per 1000 units transfused compared to 3–7 events in other national schemes suggesting underreporting (American Association of Blood Banks [AABB], 2009).

The Canadian model for haemovigilance comprises mandatory reporting for serious adverse events (SAEs) monitored by Health Canada and a voluntary reporting system for acute transfusion events through the Public Health Agency of Canada (PHAC), which is responsible for the Transfusion Transmitted Injuries Surveillance System (TTISS). PHAC reconciles, summarizes the data and publishes a report approximately 2 years after the data are collected (Ditomasso *et al*, 2012).

Data from TTISS identified a reduction in the rates of bacterial contamination for blood components and the impact of interventions to reduce these. The most significant decrease was seen following the implementation of the diversion pouch and bacterial screening of apheresis platelets (from 1 in 51 000 blood components transfused in 2003, to 1 in 360 000 by the end of 2005) (Robillard *et al*, 2011). The number of ABO incompatible, acute haemolytic transfusion reactions (AHTR), and delayed haemolytic transfusion reactions (DHTR) in Québec between 2000 and 2005 and rate of each reaction type/100 000 red cells transfused before and after the implementation of a provincial transfusion history database in the province fell, with the fall in the latter from 4·37 to 0·94, 4·62 to 2·35 and 10·79 to 4·23, respectively (Ditomasso *et al*, 2012).

### Global considerations

A World Health Organization (WHO) Global forum (November 2012) in association with the International Society of Blood Transfusion (ISBT) and the International Haemovigilance Network had representation from 46 countries. Country presentations and a survey of participants demonstrated variable development of haemovigilance schemes worldwide, hindered in many countries by lack of resources. Other challenges included fragmented blood transfusion services, cultural fear of reporting adverse incidents (blame culture/fear of retribution) and lack of government commitment and support. Even within Europe and Canada, with relatively well-developed haemovigilance systems, there are very variable reporting rates from 0·2 to 7·07 reports per 1000 units transfused (or issued to hospitals where the true denominator data is not gathered) (Robillard, 2008) and 0 to 6·8 from participating hospitals in the UK (Bolton-Maggs & Cohen, 2012).

## The Serious Hazards of Transfusion scheme (SHOT)

Reporting to SHOT started in 1996. Hospitals registered with the UK National External Quality Assurance Scheme (UK NEQAS) for blood transfusion laboratory practice were invited to participate and the scheme was widely advertised at meetings and with a leading article in the British Medical Journal (Williamson *et al*, 1996). To report an incident, the hospital clinician (usually a consultant haematologist), contacted the SHOT office, and was sent a questionnaire appropriate to the type of event. The idea of reporting when things go wrong was relatively new at this time and while the need to report transfusion-transmitted infection and other immunological complications was clear, hospital staff were more anxious about reporting events related to error. The data are confidential, and individual patient details are not included. The adverse events are reported in different categories that over the years have been expanded and modified (Table 4). Reports of errors with anti-D immunoglobulin in obstetric practice were not originally requested but are now included (from 2007) because several reports were received in the early years. A key finding from these data is that most errors are late or missed prophylactic doses of anti-D immunoglobulin, putting women at risk. Most of these errors are made by midwives and nurses (Bolton-Maggs *et al*, 2013b). ‘Near miss’ incidents were collected from 2000/2001. Handling and storage errors, and a category for inappropriate and unnecessary transfusions, were grouped separately from 2002. Adverse incidents from autologous transfusion or cell salvage were recorded from 2007, transfusion-associated circulatory overload was analysed as a separate category from 2007 and transfusion-associated dyspnoea from 2008, reflecting the evolving definitions of pulmonary complications of transfusion. Paediatric cases have been systematically analysed since 2007 after recognition that children are at increased risk compared to adults, particularly for missed specific requirements. Undertransfusion or delays have been systematically collected since 2008. Since 2010, reports are made through an online database.

**Table 4:** SHOT reporting categories

Original reporting categories 1996	Expanded reporting categories 2012
Incorrect blood or component transfused (IBCT)	IBCT includes wrong component transfused or one where specific requirements were not met, and errors related to information technology from 2007
Acute transfusion reactions (all, including haemolytic reactions occurring within 24 h)	Acute transfusion reactions (allergic, hypotensive and severe febrile)
Delayed haemolytic transfusion reactions (>24 h post-transfusion)	Haemolytic transfusion reactions (acute or delayed) Alloimmunization (separated out in 2012 and optional)
Transfusion-related acute graft-versus-host disease	
Transfusion-related acute lung injury	
Post-transfusion purpura	Post-transfusion purpura with addition of platelet transfusions as possible cause from 2012
Bacterial infection	Transfusion-transmitted infection
Viral infection	
Other infection		

	Transfusion-associated circulatory overload
	Transfusion-associated dyspnoea
	Adverse incidents related to autologous transfusion and cell salvage
	Avoidable, delayed or undertransfusion (formerly inappropriate and unnecessary transfusion, introduced in 2000)
	Handling and storage errors from 2000
	Right blood, right patient
	Anti-D errors
	Near miss events

In the first year of reporting, 1996/7, a total of 169 reports was received from 94 (22·1%) of the potential 424 hospital sites. Year on year there has been a progressive increase to almost universal participation with 99·5% of all National Health Service (NHS) hospitals, Trusts and Health Boards registered with the SHOT scheme in 2012, and 97·8% of these submitted reports. This increased participation is encouraging, but there is a wide variation in the number of reports per hospital, even when the usage is taken into consideration (estimated by the number of components issued to the hospital). The number of reports ranges from none to 6·8 per 1000 components issued. The overall number of reports submitted to SHOT per annum is now over 3500; some of these are withdrawn because the event does not qualify for inclusion or because the report is not completed within 6 months of submission.

Although reporting was initially voluntary, it is now supported by healthcare standards in England, Scotland and Wales (Department of Health, 1998, 2002, 2007; Dabaghian *et al*, 2004; NHS Quality Improvement Scotland, 2006; http://www.nhswalesgovernance.com/Uploads/Resources/Mgl7tpOP1.pdf), by the National Patient Safety Agency (NPSA) (NPSA, 2006, 2009) and mandated by several professional organizations, including Clinical Pathology Accreditation (CPA, 2009) and the General Medical Council (GMC). Doctors are required to report adverse outcomes and to participate in confidential enquiries as instructed in the recently revised advice (GMC, 2013).

**Figure 1:**
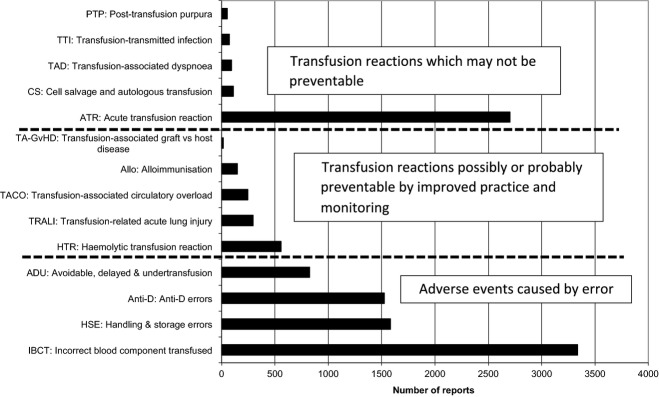
Cumulative data for SHOT categories 1996/7 to 2012, n 11570. Reported events can be divided into three groups: those caused by error that should be preventable, those caused by unpredictable reactions, and an intermediate group of complications that may be preventable by better pretransfusion assessment and monitoring.

**Figure 2:**
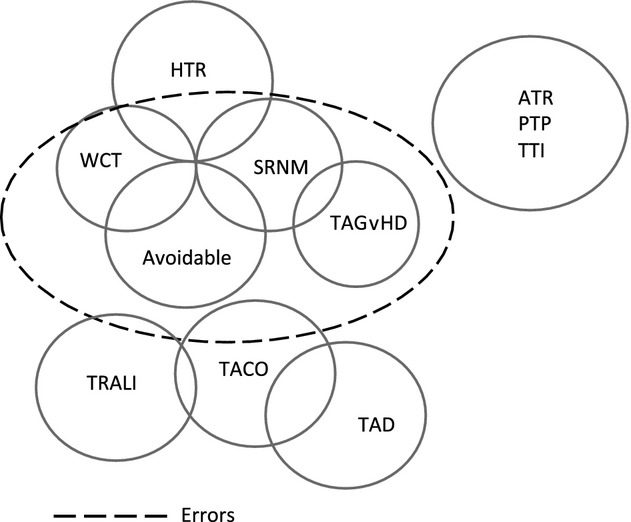
Venn diagram showing interrelationships between different adverse incidents following blood transfusion. HTR, Haemolytic transfusion reaction; TTI, Transfusion-transmitted infection; PTP, Post-transfusion purpura; TACO, Transfusion-associated circulatory overload; TAD, Transfusion-associated dyspnoea; TRALI, Transfusion-related acute lung injury; TAGvHD, Transfusion-associated graft versus host disease; SRNM, Specific requirement not met; WCT, Wrong component transfused. Avoidable: transfusions that are unnecessary or given on the basis of wrong blood results. Examples: A wrong component transfusion, e.g. group A red cells transfused to a group O patient is likely to cause a haemolytic transfusion reaction (HTR). A patient with sickle cell disease may have an HTR if transfused with red cells not matched for an antigen that previously has been associated with an antibody in that patient. A patient inappropriately transfused for a wrong haemoglobin result may develop TACO. Symptoms of TACO may overlap with other pulmonary categories, TRALI or TAD. A patient with immune deficiency who receives red that which are not irradiated (specific requirements not met) may develop TAGvHD.

Cumulative analysis of all reports (Fig 1) shows that the most common unpredictable transfusion reaction is an acute transfusion reaction (allergic, severe febrile or anaphylactic). However, overall the most common adverse incidents are caused by errors, resulting in the transfusion of an incorrect component or one that does not meet the specific requirements of the patient (e.g. not irradiated or not appropriately antigen matched). Figure 2 shows how errors relate to transfusion reactions including some pulmonary complications, particularly transfusion-associated circulatory overload, and to some haemolytic reactions, for example, by failure to provide appropriately phenotyped units to people with sickle cell disease, but others are not preventable. Kidd alloantibodies (anti-Jk^a^) are the most frequently implicated in haemolytic transfusion reactions and may be the most difficult to detect, while the pattern observed in alloimmunization without clinical reaction is different, most commonly anti-E and anti-K (Bolton-Maggs *et al*, 2013a).

## Achievements of shot

### SHOT outputs and changes in transfusion practice

SHOT publishes annual reports (see http://www.shotuk.org) and holds an annual one-day symposium where key findings and relevant transfusion issues are discussed. In 16 years of reporting the findings have triggered changes in practice at the Blood Services to reduce complications (e.g. bacterial infections and TRALI). SHOT evidence of wrong transfusions and other mistakes resulted in several national initiatives to improve training and safety that are discussed in a subsequent section.

## Reduction of unpredictable transfusion reactions

### Reduction in bacterial infections

Bacterial infections from transfusion are rare, but 24 (six fatal) were observed from 1996 to 2002. Most were related to platelet transfusions and caused by skin flora. Clinicians perhaps assumed these to be central line infections in neutropenic patients undergoing chemotherapy. This emergence of this pattern from SHOT reporting led to the recommendation that strategies be developed to reduce this. Similar observations were made in France and elsewhere (Wagner, 2004). The Blood Services introduced diversion of the first 20 ml of each donation in 2002, and also methods to improve skin cleansing, which have to be meticulously performed. In addition, screening of all platelet donations for bacterial contamination has also been introduced more recently. Fewer bacterial infections were reported in the following years, and none since 2010.

### Reduction in transfusion-related acute lung injury (TRALI)

Serial review of cases of TRALI from 1996 to 2003 demonstrated that the risk was greatest for plasma-rich components. The highest risk was from platelets and fresh frozen plasma (7–8-fold increased risk compared to red cells) and the lowest from red cells. Female donors were implicated in these cases. SHOT recommended in the 2000/2001 report (Love *et al*, 2002) that exclusion of female donors be considered in relation to the plasma used to suspend pooled platelet concentrates. The Blood Services introduced risk-reducing strategies, such as the move to male donors for fresh frozen plasma in 2003, and for suspension of platelet pools, and preferential recruitment of male apheresis platelet donors. Newly recruited female platelet donors are screened for HLA/HNA antibodies and retested after pregnancies. With the introduction of these strategies, the number of TRALI cases has decreased from a peak of 36 suspected cases (seven deaths) in 2003 to 11 suspected cases (no deaths) in 2012 (Bolton-Maggs *et al*, 2013a).

### Reduction in transfusion-associated graft-versus-host disease (TAGvHD) and post-transfusion purpura (PTP)

SHOT data provide an opportunity to confirm the beneficial effects of other strategies. For example, despite 877 reports where patients at risk had not received irradiated components in the past 11 years, there have been almost no cases of TAGvHD when patients were transfused with leucodepleted components (introduced in 1999). The last case was reported in 2000/2001 (Love *et al*, 2002). This strongly suggests that leucodepletion reduces (but does not eliminate) the risk (Williamson *et al*, 2007). Two cases of TAGvHD were recently reported when fresh non-irradiated non-leucodepleted blood was given, one in the USA in a military setting (Gilstad *et al*, 2012). The second case occurred after maternal blood was given for an emergency intrauterine transfusion and is fully discussed in the Annual SHOT Report 2012 (Bolton-Maggs *et al*, 2013a). The number of reported cases of PTP also showed a very noticeable reduction after the introduction of leucodepletion, from 9 to 11 cases per year to 1–3 since 2000.

## Surveillance of transfusion incidents flags up emerging issues

### Transfusion-associated circulatory overload (TACO)

TACO has been reported as a separate SHOT category since 2007. The ISBT set out criteria for diagnosis in 2006 (Popovsky *et al*, 2006). These include the development of any four of the following signs within 6 h of transfusion — acute respiratory distress, tachycardia, increased blood pressure, acute or worsening pulmonary oedema and evidence of positive fluid balance. TACO cases are responsible for most deaths and major morbidity reported to SHOT, with an increasing number reported year on year. TACO is the second most common cause of transfusion-related deaths (18%) reported to the FDA for the USA (Table 3), but over a 5-year period there were only 35 cases. This suggests considerable under-reporting because there have been 24 deaths from TACO reported to SHOT in the last 5 years, and 88 cases of major morbidity. Cases with pulmonary symptoms may be difficult to classify and reporters are encouraged to complete a pulmonary questionnaire to assist case allocation. SHOT data show that the majority, but not all occur in the elderly (57% over 70 years of age), but pregnant women are at risk and cases have been reported in infants. TACO may occur after relatively small amounts of blood or other components.

Overtransfusion is an avoidable precipitating factor, and it is clear that the ‘rule of thumb’ that one unit of blood leads to a 10 g/l increase in haemoglobin is only applicable to adults of about 70 kg body weight. The prescription for low body weight individuals should be reduced, and a volume of 4 ml/kg will typically give a haemoglobin increment of 10 g/l. It is of note that the recent audit of transfusions given to medical patients has confirmed an increasing haemoglobin increment per unit transfused in patients with lower body weight. Additional risk factors are recognized, particularly cardiac or renal impairment. A recent case controlled study has confirmed these risk factors (Murphy *et al*, 2013) and although there were age differences, with 67·5% over 60 years of age in the TACO group compared with 47·2% aged over 60 years in the controls, age did not emerge as a risk factor in the multivariate analysis. Careful assessment prior to transfusion is essential. SHOT reports show that many of these patients receive a unit of red cells infused over 2–3 h and this in part may be related to daycase management, with the need to complete the transfusion of 3–4 units in a day. Additional guidance was published as an addendum to the British Committee for Standards in Haematology (BCSH) guidelines on the administration of blood components (BCSH, 2010, 2012).

### Necrotizing enterocolitis in infancy (NEC)

There is evidence of an association between NEC and blood transfusion in infants but the mechanism is unclear (Mohamed & Shah, 2012; Stritzke *et al*, 2013). SHOT received two case reports in 2011 (one fatal), but none in 2012. A national UK study of NEC outcomes, including transfusion data, may help to clarify the relationship (March 2013-February 2014, see http://www.npeu.ox.ac.uk/baps-cass/surveillance/ne).

### Avoidable, delayed or undertransfusion

Transfusions are not always given appropriately. This may be due to wrong haemoglobin results, failure to assess patients appropriately, or avoidable use of emergency O RhD negative units because of poor communication or planning. One notable example, a tendency to overtransfuse patients with gastrointestinal bleeding, led to recommendations for more frequent monitoring of the haemoglobin level after every 2–3 units (Knowles & Cohen, 2011). This is reinforced by the recent National Institute for Health and Care Excellence (NICE) guidelines for management of gastrointestinal bleeding (NICE, 2012), the results of the National Comparative Audit of the medical use of red cells (K. Pendry, Consultant Haematologist, Manchester Blood Centre, Manchester, personal communication), and a randomized study of liberal *versus* restrictive transfusion in patients with severe upper gastrointestinal bleeding that demonstrated improved outcomes in the restrictive group (Villanueva *et al*, 2013).

### Complications in patients with sickle cell disease

Review of cases of haemolytic transfusion reactions (HTR) shows an over-representation of patients with sickle cell disease (SCD). These cases are described separately in SHOT reports for 2011 (Bolton-Maggs & Cohen, 2012) and 2012 (Bolton-Maggs *et al*, 2013a) because HTR are associated with major morbidity (10 of 16 cases in sickle cell patients over three years) and death (a child in 2010). Some of these reactions could be prevented by better communication between clinical teams and the transfusion laboratory (informing the laboratory of the diagnosis of SCD). SCD patients are at particular risk of alloimmunization, which can be reduced by red cell phenotyping prior to the first transfusion followed by routine matching for at least the Rh and Kell groups (Vichinsky *et al*, 2001). However this has been challenged by a recent study that showed no difference in alloimmunization rates between centres in the United States that provided closer antigen matching compared to those who did not (Miller *et al*, 2013). Many patients with SCD attend more than one hospital and there is a need to develop improved inter-laboratory communication about historical antibody and blood group information. The NHS Blood and Transplant reference laboratories are implementing electronic reporting that can be accessed by hospital transfusion laboratories; this could make it possible to share data on complex patients (Specialist Services Electronic Reporting using Sunquest ICE). A revision to the Caldicott guidelines (Department of Health, 2013) notes that ‘the duty to share information can be as important as the duty to protect patient confidentiality’.

## Development of strategies to improve transfusion safety

**Figure 3:**
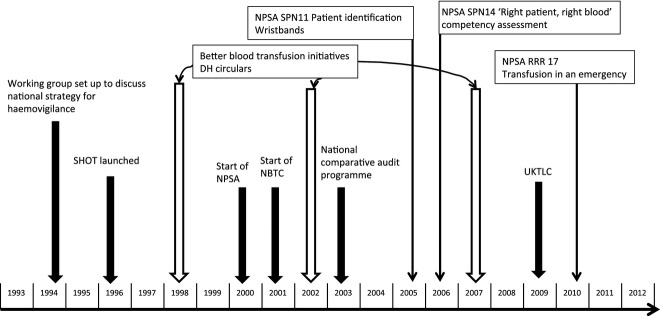
Timeline for SHOT development showing organizations that SHOT reporting has triggered or supported. SHOT, Serious Hazards of Transfusion; NPSA, national patient safety agency; SPN, Safer practice notice; RRR, Rapid response report; NBTC, National blood transfusion committee; UKTLC, UK transfusion laboratory collaborative.

Data gathered by SHOT reporting has underpinned the development of several strategies to improve transfusion safety and a timeline is shown in Fig 3.

### Guidelines for improving practice

The outstanding finding every year from SHOT reporting is that incorrect blood component transfusions make up the largest group of adverse incidents. The most serious of these are ABO-incompatible red cell transfusions resulting in death or major morbidity. One strategy for improvement is the continued development of BCSH guidelines (and addenda) on all aspects of transfusion practice (29 were produced up to 1996, and a further 24 to date). The first handbook of transfusion medicine was produced in 1989 and made available to hospital staff. It is now in the 4th edition and available at http://www.transfusionguidelines.org.uk/. In addition, a comedy training video was produced in 2002, that demonstrates how many types of error occur, and the many different people involved in transfusion. It is available as a download from Youtube or can be ordered from NHS Blood and Transplant (http://hospital.blood.co.uk/training/penny_allison/).

### National transfusion audit programme

Reporting to SHOT has the disadvantage of any confidential enquiry; the absence of true denominator data both for numbers of patients transfused (components issued to hospitals is used as a reasonable surrogate). In addition, the reporting rates vary considerably, even between hospitals with similar issue data. In 2002 SHOT recommended that basic epidemiological research was needed in the transfusion process (Love *et al*, 2002). In response, the National Comparative Audit of Blood Transfusion programme was set up in association with the Royal College of Physicians (http://hospital.blood.co.uk/safe use/clinical audit/national comparative/index.asp), producing its first report in 2003. This examined hospital transfusion practice in England, and specifically the use of wristbands during transfusion, observations during transfusion, and hospital transfusion policies in relation to BCSH guidelines. The national audits are very valuable in providing denominator data and assessment of adherence to transfusion guidelines.

Where audits have been repeated, progressive improvements in standards can be shown, for example the 2011 re-audit of bedside administration (the third audit) showed an improvement in the numbers of patients wearing wristband at the time of transfusion and better monitoring (National Comparative Audit, 2011). Hospitals can see their own data in comparison other hospitals in their region. Overall, the percentage of patients wearing a wristband had increased from 86% in the first audit (2003) to 99·5% in the 2011 audit. The recent audit shows that there are still problems, particularly in paediatrics where children and neonates were less likely to be wearing wristbands (absent in 9·5% and 12·9% respectively) than adults (absent in 1·8%).

### Better blood transfusion initiatives

The first years of SHOT confirmed that patients were more at risk of a wrong transfusion than of any other transfusion reaction. A co-ordinated national approach was required to address this. A symposium on ‘evidence-based blood transfusion’ was held by the UK Chief Medical Officers (CMO) in July 1998 and was followed by the publication of the first ‘Better Blood Transfusion; (BBT) Health Service Circular (1998/224) (Department of Health, 1998). This required all NHS Trusts where blood is transfused to ensure that multidisciplinary transfusion committees were in place and participation in the annual SHOT enquiry. In addition, recommendations were made about education and training. Further circulars followed in 2002 and 2007 (Department of Health, 2002, 2007). The third focused on safe and appropriate use of blood. These gave additional support for the development of the Hospital Transfusion Practitioner role. These individuals have been vital in teaching and training and collection of data for the national audits. Not surprisingly there have been constraints on achieving these recommendations.

The CMO's National Blood Transfusion Committee (NBTC) performed regular surveys of progress. The most recent (Murphy *et al*, 2010) noted that the key factors preventing implementation were inadequate staff for the hospital transfusion team and transfusion not being a high enough priority for Trusts and Strategic Health Authorities. Compliance with BSQR (2005) and the requirements for competency assessments from the NPSA Safer Practice Notice 14 (NPSA, 2006) placed competing demands on the transfusion teams. ‘Patient blood management’ is a new initiative, ‘a multidisciplinary, evidence-based approach to optimizing the care of patients who might need blood transfusion’ introduced in 2012 (NBTC, 2013).

### The national patient safety agency (NPSA)

The NPSA was set up by the Department of Health in 2001 as a special Health Authority with a mandate to improve patient safety by establishing and managing a national reporting and learning system (NLRS) for incidents that affect patient safety. This followed publication of the report ‘An Organisation with a Memory’ (Department of Health, 2000) and the need to encourage a more open culture to enable reporting when things go wrong. The NLRS collates information on incidents across the NHS. In partnership with SHOT and the NBTC, the NPSA published two relevant alerts, SPN14, Right Patient, Right Blood (NPSA, 2006), and SPN 002, Risk to patient safety of not using the NHS number (NPSA, 2009). A Rapid Response Report (NPSA, 2010) ‘the transfusion of blood and blood components in an emergency’ (RRR017) responded to deaths occurring from delays in provision of blood. RRR017 required that all such delays be reported to SHOT and the NPSA. The NPSA promoted root cause analysis as a means to learn from the incidents. The NPSA key functions were transferred to the NHS commissioning Board Special Health Authority from June 2012 and it remains to be seen how effectively ‘patient safety’ measures will be driven under the new commissioning arrangements. The ‘never events’ policy was introduced to the NHS in 2009 following a recommendation by Lord Darzi in his review of the NHS (National Health Service, 2008), and the list expanded to 25 events in 2011 (Department of Health, 2012). These are events that should never happen to patients, and they include ABO-incompatible transfusions that result in death or harm to patients, prompted by evidence from SHOT reports and SHOT's clinical lessons documents (see http://www.shotuk.org).

### The UK transfusion laboratory collaborative (UKTLC)

Serial SHOT reports demonstrated that 30–40% of wrong transfusions are caused by errors originating in hospital laboratories. The UKTLC is a collaborative group (with representation from SHOT, NBTC, the Institute of Biomedical Science (IBMS), and UK NEQAS (blood transfusion and laboratory practice)) whose purpose was to produce recommendations for minimum standards for hospital transfusion laboratories (Chaffe *et al*, 2009, 2010). The intention was to encourage appropriate levels of staffing, training and technology, with the aim of reducing laboratory errors by 50% by 30 September 2012. This reduction has not been achieved, and further surveys of laboratory staffing are being undertaken. The increasing laboratory errors are a cause for concern. The MHRA findings are similar, that most incidents are caused by human error, by skipping essential steps, rushing and distraction.

### Back to the future

How safe is transfusion today? The track record is good: the number of reports to SHOT has increased year on year, but the number of deaths and instances of major morbidity related to transfusion has decreased as a proportion of all reports, and it is clear that transfusion is very safe. However the number of errors continues to increase, and is cause for concern not least because the careful regulation of transfusion practice acts as an early warning system, flagging up dangers to patients from types of identification errors occurring elsewhere in medicine, for example medication errors (Allard *et al*, 2002; Pownall, 2009; Glavin, 2010). Data available from the NRLS showed that medication errors are common, and the second most commonly reported event by hospitals, 11% of all reports, with 149 409 reported (51 deaths, 242 severe outcomes) in the period January to December 2011 (NRLS, 2011). In addition, identification errors leading to wrong blood samples may have impact on other pathology areas. Wrong biochemistry or microbiology results can lead to unnecessary and possible harmful interventions for the wrong patient. Each year, an average of 250 wrong blood incidents are reported to SHOT but, fortunately, very few are ABO-incompatible transfusions (about 10 each year). Each one of these is potentially lethal. The SHOT report 2009 (Taylor *et al*, 2010) recommended a patient education campaign to empower patients to ask ‘do you know who I am?’ before any intervention, and the campaign was launched in 2012 with materials available on the NBTC website (NBTC, 2012).

The contributory factors are similar in all areas of medical practice where humans are involved. These are slips and lapses, taking short cuts, distractions, and omission of essential steps. These ‘human factors’ are widely recognized (Keogh, 2012), but the more difficult question is how can this be changed? Removal of manual steps in transfusion has improved transfusion safety, from the introduction of automated analysers in laboratories to electronic systems across the complete transfusion process, including bar-code readers at the bedside leading to end-to-end electronic control (Davies *et al*, 2006).

‘Near miss’ events make up a third of all SHOT reports, about 70% of these are clinical errors, including approximately 100 wrong blood samples caught for every one that results in a wrong component transfusion. The majority of sample errors result from failure to identify the patient correctly. More can be done to encourage hospital transfusion staff to learn from these incidents. Root cause analysis is a very useful tool in incident review and is the piece most often missing from SHOT reports; a chapter on this is therefore included in the 2012 report (Bolton-Maggs *et al*, 2013a).

It is not easy for reporters to have two reporting systems. In the past 12 months the MHRA and SHOT have worked together towards development of a single haemovigilance system which will encompass both the requirements of EU legislation without detracting from the more detailed clinical event reporting required by SHOT. A single haemovigilance system would have significant benefits both for hospital reporters and national and international regulation.

Further work is required to improve transfusion education and training, both clinically and in the laboratory and areas for further research have been identified (Heddle *et al*, 2012). Most errors relate to the failure of simple standard procedures so that the current emphasis is on ‘back to basics’ in 2012, and renewed focus on correct patient identification in 2013. This was the key recommendation in the first SHOT report (Love & Lowe, 1998). SHOT has recommended the introduction of a transfusion checklist because this has proved effective in reducing errors in surgery (Haynes *et al*, 2009; Bolton-Maggs & Cohen, 2012).

Could transfusion become less safe in the future? Changes in medical staffing, such as the difficulty recruiting into general medicine reported by the Royal College of Physicians (RCP, 2012; RCP, 2013) and accident and emergency medicine together with major restructuring of laboratory services; pathology modernization resulting in laboratory mergers, reduction in senior staff, de-skilling and job insecurity, are all cause for concern.

There is no doubt that the SHOT scheme has contributed to transfusion safety in many ways. Although confidential enquiries have been criticized for having no denominator data, little evidence of benefit and working from anecdotal reports (Angelow & Black, 2011), it is clear that the output from SHOT has made a major contribution to transfusion safety. The data are increasingly robust with the virtually complete participation across the UK and surrogate denominator data are indicated by the number of components issued to hospitals. There is a clear need for continued vigilance.

## Key learning points

1. Transfusion is very safe in the UK with a very low risk of bacterial or viral infection
2. ‘Back to basics’: correct patient identification and adherence to basic procedures are key to safer practice
3. Errors continue to make up half or more of all events reported to SHOT
4. Acute transfusion reactions are the now the leading cause of major morbidity
5. Transfusion-associated circulatory overload is a serious complication (43% of reported cases resulted in death or major morbidity in 2012)
6. Adverse event reporting and investigation has resulted in changes in transfusion practice which have reduced pathological reactions, for example bacterial infections and TRALI

## References

[b1] Allard J, Carthey J, Cope J, Pitt M, Woodward S (2002). Medication errors: causes, prevention and reduction. British Journal of Haematology.

[b2] American Association of Blood Banks (AABB) (2009). http://www.aabb.org/programs/biovigilance/nbcus/Documents/09-nbcus-report.pdf.

[b3] Angelow A, Black N (2011). The use and impact of national confidential enquiries in high-income countries. British Medical Journal Quality and Safety.

[b4] BCSH (2010). http://www.bcshguidelines.com/documents/Admin_blood_components_bcsh_05012010.pdf.

[b5] BCSH (2012). http://www.bcshguidelines.com/documents/BCSH_Blood_Admin_-_addendum_August_2012.pdf.

[b6] Bolton-Maggs P, Poles D, Watt A, Cohen H, Thomas D, on behalf of the SHOT Steering Group (2013a). http://www.shotuk.org/wp-content/uploads/2013/08/SHOT-Annual-Report-2012.pdf.

[b7] Bolton-Maggs PHB, Cohen H, on behalf of the SHOT Steering Group (2012). http://www.shotuk.org/wp-content/uploads/2012/07/SHOT-ANNUAL-REPORT_FinalWebVersionBookmarked_2012_06_22.pdf.

[b8] Bolton-Maggs P, Davies T, Poles D, Cohen H (2013b). Errors in anti-D immunoglobulin administration: retrospective analysis of 15 years of reports to the UK confidential haemovigilance scheme. British Journal of Obstetrics and Gynaecology.

[b9] BSQR (2005). http://www.legislation.gov.uk/uksi/2005/2898/pdfs/uksi_20052898_en.pdf.

[b10] Chaffe B, Jones J, Milkins C, Taylor C, Asher D, Glencross H, Murphy M, Cohen H (2009). UK Transfusion Laboratory Collaborative: recommended minimum standards for hospital transfusion laboratories. Transfusion Medicine.

[b11] Chaffe B, Jones J, Milkins C, Taylor C, Asher D, Glencross H, Murphy M, Cohen H (2010). Recommended minimum standards for hospital transfusion laboratories - UK Transfusion Laboratory Collaborative. Biomedical Scientist.

[b12] Clinical Pathology Accreditation (CPA) (2009). http://www.cpa-uk.co.uk/files/PD-LAB-Standards_v2.02_Nov_2010.pdf.

[b13] Dabaghian RH, Mortimer PP, Clewley JP (2004). Prospects for the development of pre-mortem laboratory diagnostic tests for Creutzfeldt-Jakob disease. Reviews in Medical Virology.

[b14] Davies A, Staves J, Kay J, Casbard A, Murphy MF (2006). End-to-end electronic control of the hospital transfusion process to increase the safety of blood transfusion: strengths and weaknesses. Transfusion.

[b15] Department of Health (1998). http://webarchive.nationalarchives.gov.uk/20130107105354/http://www.dh.gov.uk/en/Publicationsandstatistics/Lettersandcirculars/Healthservicecirculars/DH_4004262.

[b16] Department of Health (2000). http://webarchive.nationalarchives.gov.uk/+/www.dh.gov.uk/en/Publicationsandstatistics/publications/publicationspolicyandguidance/browsable/dh_4098184.

[b17] Department of Health (2002). http://webarchive.nationalarchives.gov.uk/20130107105354/http://www.dh.gov.uk/en/Publicationsandstatistics/Lettersandcirculars/Healthservicecirculars/DH_4004264.

[b18] Department of Health (2007). http://www.transfusionguidelines.org/docs/pdfs/nbtc_bbt_hsc_07.pdf.

[b19] Department of Health (2012). https://www.gov.uk/government/uploads/system/uploads/attachment_data/file/215206/dh_132352.pdf.

[b20] Department of Health (2013). http://caldicott2.dh.gov.uk/.

[b21] Ditomasso J, Liu Y, Heddle NM (2012). The Canadian Transfusion Surveillance System: what is it and how can the data be used?. Transfusion and Apheresis Science.

[b22] European Commission (2003). Directive 2002/98/EC of the European Parliament and of the Council of 27 January 2003, setting standards of quality and safety for the collection, testing, processing, storage and distribution of human blood and blood components and amending Directive 2001/83/EC. L33. Official Journal of the European Union.

[b23] European Commission (2005). Directive 2005/61/EC of 30 September 2005 implementing Directive 2002/98/EC of the European Parliament and of the Council as regards traceability requirements and notification of serious adverse reactions and events (Text with EEA relevance). L256. Official Journal of the European Union.

[b24] General Medical Council (2013). http://www.gmc-uk.org/static/documents/content/GMP_2013.pdf_51447599.pdf.

[b25] Gilstad C, Roschewski M, Wells J, Delmas A, Lackey J, Uribe P, Popa C, Jardeleza T, Roop S (2012). Fatal transfusion-associated graft-versus-host disease with concomitant immune hemolysis in a group A combat trauma patient resuscitated with group O fresh whole blood. Transfusion.

[b26] Glavin RJ (2010). Drug errors: consequences, mechanisms, and avoidance. British Journal of Anaesthetics.

[b27] Haynes AB, Weiser TG, Berry WR, Lipsitz SR, Breizat AH, Dellinger EP, Herbosa T, Joseph S, Kibatala PL, Lapitan MC, Merry AF, Moorthy K, Reznick RK, Taylor B, Gawande AA, Safe Surgery Saves Lives Study Group (2009). A surgical safety checklist to reduce morbidity and mortality in a global population. New England Journal of Medicine.

[b28] Heddle NM, Fung M, Hervig T, Szczepiorkowski ZM, Torretta L, Arnold E, Lane S, Murphy M (2012). Challenges and opportunities to prevent transfusion errors: a Qualitative Evaluation for Safer Transfusion. Transfusion.

[b29] International Haemovigliance Network (IHN) (2012). http://www.ihnorg.com/about/definition-of-haemovigilance/.

[b30] Keogh B (2012). http://www.chfg.org/news-blog/dh-human-factors-group-interim-report-and-recommendations-for-the-nhs.

[b31] Knowles S, Cohen H (2011). http://www.shotuk.org/wp-content/uploads/2011/10/SHOT-2010-Report1.pdf.

[b32] Love E, Lowe S (1998). http://www.shotuk.org/wp-content/uploads/2010/03/SHOT-Report-96-97.pdf.

[b33] Love E, Soldan K, Jones H (2002). http://www.shotuk.org/wp-content/uploads/2010/03/SHOT-Report-00-01.pdf.

[b34] McClelland DB (1998). Treating a sick process. Transfusion.

[b35] Miller ST, Kim H-Y, Weiner DL, Wager CG, Gallagher D, Styles LA, Dampier CD, Roseff SD (2013). Red blood cell alloimmunisation in sickle cell disease: prevalence in 2010. Transfusion.

[b36] Mohamed A, Shah PS (2012). Transfusion-associated necrotizing enterocolitis: a meta-analysis of observational data. Pediatrics.

[b37] Murphy M, Gerrard R, Howell C, Little T (2010). http://www.transfusionguidelines.org.uk/docs/pdfs/nbtc_bbt_survey_2010v5.pdf.

[b38] Murphy EL, Kwaan N, Looney MR, Gajic O, Hubmayr RD, Gropper MA, Koenigsberg M, Wilson G, Matthay M, Bacchetti P, Toy P, Group TS (2013). Risk factors and outcomes in transfusion-associated circulatory overload. American Journal of Medicine.

[b39] National Comparative Audit (2011). http://hospital.blood.co.uk/library/pdf/NCA_2011_bedside_tx_re-audit_report.pdf.

[b40] National Health Service (2008). http://webarchive.nationalarchives.gov.uk/20130107105354/http://www.dh.gov.uk/en/Publicationsandstatistics/Publications/PublicationsPolicyAndGuidance/DH_085825.

[b41] NBTC (2012). http://www.transfusionguidelines.org/index.aspx?pageid=982&section=27&publication=NTC.

[b42] NBTC (2013). http://www.transfusionguidelines.org.uk/Index.aspx?Publication=NTC&Section=27&pageid=7728.

[b43] NHS Quality Improvement Scotland (2006). http://www.healthcareimprovementscotland.org/previous_resources/standards/blood_transfusion.aspx.

[b44] NICE (2012). http://www.nice.org.uk/nicemmedia/live/13762/59549/59549.pdf.

[b45] NPSA (2006). http://www.nrls.npsa.nhs.uk/EasySiteWeb/GatewayLink.aspx?alId=60046.

[b46] NPSA (2009). http://www.nrls.npsa.nhs.uk/resources/type/alerts/?entryid45=61913&p=2.

[b47] NPSA (2010). http://www.nrls.npsa.nhs.uk/EasySiteWeb/getresource.axd?AssetID=83688.

[b48] NRLS (2011). http://www.nrls.npsa.nhs.uk/resources/type/data-reports/?entryid45 = 135152.

[b49] Popovsky MA, Robillard P, Schipperus M, Stainsby D, Tissot JD, Wiersum J (2006). http://www.ihn-org.com/wp-content/uploads/2011/06/ISBT-definitions-for-non-infectious-transfusion-reactions.pdf.

[b50] Pownall M (2009). Complex working environment, not poor training, blamed for drug errors. British Medical Journal.

[b51] Public Health England (2013). http://www.hpa.org.uk/Topics/InfectiousDiseases/InfectionsAZ/BIBD/EpidemiologicalData/bibd020EstfreqofinfecteddonenteringUKbloodsupply/.

[b52] Robillard P (2008). http://www.ihn-org.com/wp-content/uploads/2011/02/Robillard-Pierre-21.pdf.

[b53] Robillard P, Delage G, Nawej KI, Goldman M (2011). Use of hemovigilance data to evaluate the effectiveness of diversion and bacterial detection. Transfusion.

[b54] Royal College of Physicians (2012). http://www.rcplondon.ac.uk/sites/default/files/documents/hospitals-on-the-edge-report.pdf.

[b55] Royal College of Physicians (2013). http://www.rcplondon.ac.uk/sites/default/files/hospital-workforce-fit-for-the-future1.pdf.

[b56] Stritzke AI, Smyth J, Synnes A, Lee SK, Shah PS (2013). Transfusion-associated necrotising enterocolitis in neonates. Archives of Disease in Childhood. Fetal and neonatal edition.

[b57] Taylor C, Cohen H, Mold D, Jones H (2010). http://www.shotuk.org/wp-content/uploads/2010/07/SHOT2009.pdf.

[b58] TRIP (Transfusion Reactions in Patients) Annual Report (2011). http://www.tripnet.nl/pages/en/documents/TRIP2011HemovigilanceExtendedversion.pdf.

[b59] U.S Food and Drug Administration (FDA) (2013). http://www.fda.gov/BiologicsBloodVaccines/SafetyAvailability/ReportaProblem/TransfusionDonationFatalities/ucm346639.htm.

[b60] Vichinsky EP, Luban NL, Wright E, Olivieri N, Driscoll C, Pegelow CH, Adams RJ (2001). Prospective RBC phenotype matching in a stroke-prevention trial in sickle cell anemia: a multicenter transfusion trial. Transfusion.

[b61] Villanueva C, Colomo A, Bosch A, Concepcion M, Hernandez-Gea V, Aracil C, Graupera I, Poca M, Alvarez-Urturi C, Gordillo J, Guarner-Argente C, Santalo M, Muniz E, Guarner C (2013). Transfusion strategies for acute upper gastrointestinal bleeding. New England Journal of Medicine.

[b62] Wagner SJ (2004). Transfusion-transmitted bacterial infection: risks, sources and interventions. Vox Sanguinis.

[b63] Williamson LM, Heptonstall J, Soldan K (1996). A SHOT in the arm for safer blood transfusion. British Medical Journal.

[b64] Williamson LM, Stainsby D, Jones H, Love E, Chapman CE, Navarrete C, Lucas G, Beatty C, Casbard A, Cohen H (2007). The impact of universal leukodepletion of the blood supply on hemovigilance reports of posttransfusion purpura and transfusion-associated graft versus-host disease. Transfusion.

